# Identification of prognosis and therapy related intratumoral microbiome and immune signatures in gastric cancer

**DOI:** 10.3389/fimmu.2025.1622959

**Published:** 2025-07-03

**Authors:** Peng Jin, Xiaoyan Ji, Jingchao Bai, Wei Su, Bin Ke, Yong Liu, Bin Wang

**Affiliations:** ^1^ Department of Gastric Surgery, Tianjin Medical University Cancer Institute and Hospital, National Clinical Research Center for Cancer, Key Laboratory of Cancer Prevention and Therapy, Tianjin Key Laboratory of Digestive Cancer, Tianjin’s Clinical Research Center for Cancer, Tianjin, China; ^2^ Department of Emergency Ward, First Teaching Hospital of Tianjin University of Traditional Chinese Medicine, National Clinical Research Center for Chinese Medicine Acupuncture and Moxibustion, Tianjin, China; ^3^ Department of Gastrointestinal Cancer Biology, Tianjin Medical University Cancer Institute and Hospital, Key Laboratory of Cancer Immunology and Biotherapy, National Clinical Research Center for Cancer, Tianjin Medical University Cancer Institute and Hospital, Tianjin, China; ^4^ Department of Endoscopy, Tianjin Medical University Cancer Institute and Hospital, National Clinical Research Center for Cancer, Key Laboratory of Cancer Prevention and Therapy, Tianjin’s Clinical Research Center for Cancer, Tianjin, China; ^5^ Department of Pancreatic Cancer, Tianjin Medical University Cancer Institute and Hospital, National Clinical Research Center for Cancer, Tianjin Key Laboratory of Digestive Cancer, Key Laboratory of Cancer Prevention and Therapy, Tianjin, China

**Keywords:** gastric cancer, intratumoral microbiome, prognostic biomarkers, therapeutic responses, drug sensitivity, comprehensive analysis

## Abstract

The impact of the intratumoral microbiome (ITM) on the treatment and prognosis of gastric cancer (GC) remains controversial. Our study analyzed the differential ITM in GC tissues and identified nine bacterial genera significantly associated with overall survival (OS), with seven as risk factors and two as protective factors. Three distinct clusters with varying survival outcomes were defined, demonstrating correlations with pathological stage and immune features. An immune-related gene-based RiskScore model incorporating genes such as Apolipoprotein D (APOD), Stanniocalcin 1 (STC1), Coagulation Factor II Thrombin Receptor (F2R), Angiotensinogen (AGT), Fatty Acid Binding Protein 4 (FABP4), Inhibin Subunit Beta A (INHBA), Caspase Recruitment Domain Family Member 11 (CARD11), and Dickkopf WNT Signaling Pathway Inhibitor 1 (DKK1) was established and validated in The Cancer Genome Atlas (TCGA) and Gene Expression Omnibus (GEO) datasets. When combined with clinical factors, this RiskScore model formed a Nomogram model achieving Areas Under the Curve (AUCs) of 0.72, 0.76, and 0.79 for 1, 3, and 5-year OS predictions, respectively. This model exhibited robust predictive accuracy over time and correlated with mutation frequency, drug sensitivity, and immunotherapy response. Furthermore, single-cell analysis revealed that tumor-associated fibroblasts may play a pivotal role in immune-microbial interactions. The results were confirmed using quantitative real-time polymerase chain reaction (qPCR) and immunohistochemistry (IHC). In conclusion, the prognostic model incorporating ITM and immune-related genes aids in risk stratification and provides valuable insights and targets for GC treatment.

## 1 Introduction

Gastric cancer (GC) remains a significant health burden globally, with an estimated 1 million new cases and 783,000 deaths annually, making it the fifth most common malignancy and the third leading cause of cancer-related deaths worldwide (1). The burden is especially heavy in Eastern Asia, attributed to dietary, environmental, and genetic factors (2). Despite advancements in diagnostic techniques and treatment modalities, the prognosis for advanced GC remains dismal, with a 5-year survival rate of less than 30%. This poor prognosis is often due to late-stage diagnosis, high rates of metastasis, and the tumor’s complex and heterogeneous nature, presenting significant therapeutic challenges (3).

The tumor microenvironment (TME) plays a crucial role in cancer progression and patient outcomes (4). The TME comprises various cellular components, including immune cells, fibroblasts, endothelial cells, and emerging evidence highlights the significant role of the tumor-associated microbiome within the tumor and its immediate surroundings (5). The intratumoral microbiome (ITM) influences numerous physiological processes and has been implicated in the pathogenesis of various cancers (6). In particular, alterations in the microbiome have been associated with tumorigenesis through mechanisms such as chronic inflammation, immune modulation, and production of carcinogenic metabolites (7, 8) Studies have shown that specific bacterial species, such as Helicobacter pylori, play a crucial role in the development of GC by inducing chronic gastritis, leading to atrophic gastritis, intestinal metaplasia, dysplasia, and eventually carcinoma (9, 10). However, beyond H. pylori, the broader spectrum of tumor-associated microbiome and its impact on GC prognosis remains controversial (11–13). Increasing evidence indicates that the microbiome present in saliva, gastric juice, tumor tissues, and the gut are associated with the occurrence, development, treatment, and prognosis of GC (14). Recent advancements in high-throughput sequencing and bioinformatics have enabled comprehensive characterization of the microbiome, providing new insights into its role in cancer biology (7, 15).

This study aimed to analyze the differential ITM in GC tissues, assess its prognostic value, and explore immune-microbiome interactions to identify prognostic biomarkers and enhance therapeutic strategies.

## 2 Methods

### 2.1 Data collection and initial processing

GC microbiome data, along with RNA-seq and survival information, were downloaded from the TCGA database in March 2024. Preprocessing steps involved excluding genes with missing values or not expressed in >50% of samples, removing samples with >50% unexpressed genes, and log2 transforming expression values. Libraries were prepared with the TruSeq Stranded mRNA Kit (Illumina) and sequenced on an Illumina NovaSeq 6000 (150 bp paired-end). Reads were aligned to the human genome (GRCh38) using STAR (v2.7.10a). Post-preprocessing, 350 GC and paired normal samples were retained. Fresh frozen gastric cancer (GC) tissues and paired adjacent normal tissues were collected from Tianjin Medical University Cancer Institute and Hospital. The GSE62254 dataset with clinical data was downloaded from NCBI GEO, retaining 300 GC samples after excluding those with zero or missing survival data. Dataset GSE183904 was used for single-cell analysis. Additional validation samples were collected from Tianjin Medical University Cancer Institute and Hospital, with study approval (Approval No. E20210132) and informed consent from all patients.

### 2.2 Identifying prognostic microorganisms and GC subtypes

Using the limma package, we compared GC tissues with adjacent normal controls from TCGA to identify differentially abundant microorganisms. Univariate Cox regression determined which of these microorganisms were significantly associated with patient survival. From the identified prognostic microorganisms, we performed unsupervised hierarchical clustering using Consensus Cluster Plus to classify GC patients into subtypes. The optimal number of clusters was statistically determined. Survival prognosis for each cluster was assessed via Kaplan-Meier curves, and correlations with clinical factors (age, gender, stage, etc.) were investigated.

### 2.3 Immune infiltration and molecular mechanisms

Using single-sample Gene Set Enrichment Analysis (ssGSEA), we conducted immune infiltration analysis to quantify immune cell types in tumor samples. Estimation of Stromal and Immune cells in Malignant Tumors using Expression data (ESTIMATE) scores assessed the TME. Wilcoxon tests evaluated differences in immune cell proportions across clusters. Gene Set Enrichment Analysis (GSEA) and Gene Set Variation Analysis (GSVA) analyzed pathway and gene set enrichment across GC clusters.

### 2.4 Establishing and validating a prognostic risk model based on immune-related gene

Differential expression and Weighted Gene Co-expression Network Analysis (WGCNA) identified gene modules correlated with GC phenotypes. Cross-referencing these with the ImmPort database revealed immune genes linked to microorganisms. Univariate Cox regression selected genes significantly linked to patient survival. We developed a prognostic signature (Risk Score) using Least Absolute Shrinkage and Selection Operator (LASSO)-COX regression: Risk Score = β1X1 + β2X2 +… + βnXn, where β is the regression coefficient and X is the gene expression value. Patients in TCGA and GEO were categorized by their Risk Score into High- and Low-risk groups. Kaplan-Meier curves and log-rank tests confirmed the prognostic value of this signature. Incorporating clinical factors (age, gender, stage, etc) into univariate and multivariate COX regression models, we confirmed the independent prognostic value of the identified genes. We constructed a nomogram integrating these factors to aid clinicians in survival prognosis and decision-making.

### 2.5 Mutation status and immune-microbial interactions

Analyzing mutation data, we identified the top 20 most mutated genes (TOP20) and calculated Tumor Mutation Burden (TMB). Using Multiple Alignment of Fasta (maftools) and Generalized Gene Correlation analysis (ggcor) in R, we examined TMB distribution across risk groups and correlations between risk scores, microbial abundance, and immune cell populations. Differences in immune checkpoint molecule expression [PD1 (PDCD1), PD-L1 (CD274), CTLA-4 (CTLA4), CD278 (ICOS), TIM3 (HAVCR2), LAG3, CD47, BTLA, TIGIT, MYD1 (SIRPA), OX40 (TNFRSF4), 4-1BB (TNFRSF9), B7-H4 (VTCN1)] were also analyzed between risk groups.

### 2.6 Chemotherapy and immunotherapy efficacy

Using the Genomics of Drug Sensitivity in Cancer (GDSC) database and Predictive Response to Therapy (pRRophetic) package in R, we estimated chemotherapy sensitivity (IC50) and differences between risk groups. Tumor Immune Dysfunction and Exclusion (TIDE) database predicted immune checkpoint therapy responses, quantified by TIDE scores. We further analyzed Cytolytic Activity (CYT), Tertiary Lymphoid Structure (TLS) scores, and CD8A/PD-L1 ratios between risk groups using Wilcoxon tests.

### 2.7 Single-cell analysis

Based on the GC single-cell dataset GSE183904, we selected the top 2000 highly variable genes for UMAP dimensionality reduction and clustering analysis, ultimately obtaining 31 cell clusters. Cell Ranger (v6.1.2) was used for alignment and UMI counting. By referencing cell marker genes, we annotated these 31 cell clusters and successfully distinguished 8 cell types, specifically including B cells, endothelial cells (endo), epithelial cells, fibroblasts, mast cells, myeloid cells, smooth muscle cells, and T cells. Next, we compared the percentage differences of these 8 cell types between the tumor group and the normal group. Furthermore, we analyzed the expression of 8 genes in various cells within the risk model, conducted cell communication analysis, and explored the activation of ligand-receptor pairs during the interaction of different immune cells. Seurat (v4.3) for clustering and CellChat (v1.6.0) for ligand-receptor interaction analysis.

### 2.8 Validation of bioinformatics analysis results by qPCR and IHC

To validate bioinformatics analysis results, we performed qPCR and IHC experiments. For qPCR, we validated the expression levels of APOD, STC1, F2R, and AGT using 10 pairs of fresh cancerous and adjacent normal tissue samples. Total RNA was extracted, followed by cDNA synthesis and qPCR amplification using specific primers (**Supplementary Materials**). Fresh gastric cancer (GC) tissues and paired adjacent normal tissues (n = 10 pairs) were collected, snap-frozen in liquid nitrogen, and stored at −80°C. Total RNA was extracted using TRIzol Reagent, with purity (A260/A280: 1.8–2.0) and integrity verified. cDNA was synthesized from 1 µg RNA using PrimeScript RT Reagent Kit. TqRT-PCR was performed in triplicate with SYBR Green Premix (TB Green™) on a QuantStudio 5 system using gene-specific primers (APOD, STC1, F2R, AGT, ACTA2 and) and β-actin as the housekeeping gene. Relative expression (2−^ΔΔCt^ method) and statistical significance (Student’s t-test, p < 0.05) were analyzed. Conditions: 95°C for 10 min, 40 cycles of 95°C for 15 sec, 60°C for 1 min. For IHC, formalin-fixed, paraffin-embedded (FFPE) tissues were used. We conducted a co-localization analysis of STC1 and CD56 on serial sections utilizing 50 paraffin-embedded tissue samples. Formalin-fixed, paraffin-embedded (FFPE) GC and adjacent normal tissues were sectioned (4 µm), deparaffinized, and subjected to antigen retrieval (10 mM citrate buffer, pH 6.0, 95°C, 20 min). Endogenous peroxidase was blocked with 3% H_2_O_2_ (15 min, RT). These tissue sections were subsequently stained for STC1(Proteintech, 20621-1-AP, 1:200 dilution) and CD56 (Servicebio, GB12041, 1:100 dilution). Signal was developed with DAB (Dako, 5 min), counterstained with hematoxylin, and visualized under bright-field microscopy. Slides were scanned with a Leica Aperio AT2 scanner and analyzed using ImageScope (v12.4). Negative controls omitted primary antibody. Staining intensity (0–3) and percentage of positive cells were scored independently by two pathologists. The STC1 expression levels were scored, classified into high and low expression groups, and their correlation with CD56 was analyzed using Spearman’s analysis.

### 2.9 Statistical analysis

All statistical tests were performed in R (v4.3.1) with packages cited in the original manuscript. Data manipulation was conducted using dplyr and tidyr. For statistical testing, the stats package facilitated Wilcoxon and log-rank tests. Survival analyses, including Kaplan-Meier estimates and Cox models, were done using the survival package and visualized with survminer and ggplot2. Hierarchical clustering and heatmaps were generated using pheatmap. Data partitioning and model training were performed with caret, while glmnet was used for LASSO and Ridge regression. Differential expression was analyzed using limma, and additional analyses utilized Consensus Cluster Plus, ESTIMATE, cluster Profiler, GSVA, WGCNA, rms, maftools, ggcor, pRRophetic and CellChat. Graphs were created with ggplot2 and GraphPad Prism 10.0. Significant differences were set at *p* < 0.05 or *p* < 0.001.

## 3 Results

### 3.1 Prognostic intratumoral microorganisms and microbial clustering analysis

**Figure 1:**
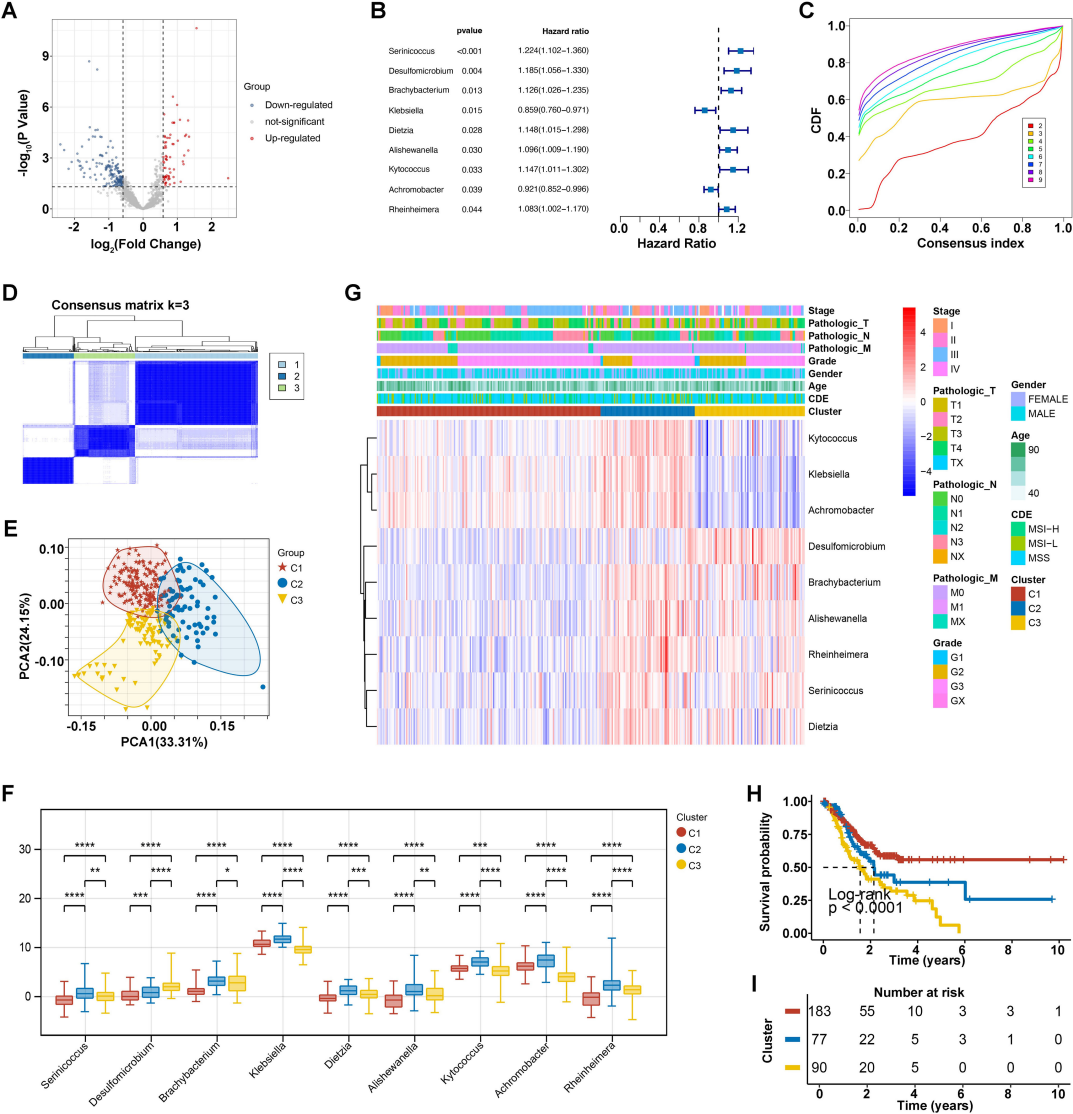
Intratumoral microorganisms and microbial clustering analysis. **(A)** Differential microbial volcano plot, where blue represents downregulated microbes and red represents upregulated microbes. **(B)** Forest plot of prognostic microbes. **(C)** Cumulative distribution function (CDF) of consensus clustering for k = 2-9. **(D)** Consensus clustering matrix for the optimal k = 3. **(E)** PCA curve analysis. **(F)** Clinical correlation heatmap of 9 prognostic bacterial genera, where red represents high expression and blue represents low expression. *p<0.05, **p<0.01, ***p<0.001, ****p<0.0001. **(G)** Differential levels of the 9 prognostic bacterial genera across different clusters. **(H, I)** KM survival curves for different clusters.

Through comparative analysis of tumor and normal samples, we identified 229 differentially expressed genera in GC: 67 upregulated and 162 down regulated (**Figure 1A**). Univariate Cox regression analysis revealed nine bacterial genera significantly associated with OS. Genera such as Serinicoccus, Desulfomicrobium, Brachybacterium, Dietzia, Alishewanella, Kytococcus, and Rheinheimera were linked to increased risk, while Klebsiella and Achromobacter correlated with decreased risk (*p* < 0.05) (**Figure 1B**).

Using these nine prognostic bacterial genera, unsupervised clustering analysis determined an optimal K value of 3, resulting in distinct microbial clusters labeled as C1, C2, and C3, comprising 183, 77, and 90 GC samples, respectively (**Figures 1C, D**). Principal component analysis (PCA) underscored the segregation among these clusters, highlighting their underlying differences (**Figure 1E**). In detail, the distribution of nine prognostic bacterial genera we have identified was analyzed across different clusters, with Kytococcus, Klebsiella and Achromobacter enriched in cluster C2 and some genera such as Desulfomicrobium and Brachybacterium was observed more in cluster C3 (**Figure 1F**). Correlation analysis with clinical data showed no significant differences in sub-type (*p* = 0.24), age (*p* = 0.26), gender (*p* = 0.31), grade (*p* = 0.24), and Pathologic N stage (*p* = 0.06) across three clusters. However, microbial clusters significantly correlated with Pathologic M (*p* =0.01), Pathologic T (*p* < 0.0001), and Stage (*p* < 0.000) (**Table 1**). The differential levels of the 9 prognostic bacterialgenera across different clusters was shown in **Figure 1G**. Theatmap analysis revealed distinct microbial composition patterns across tumor stages, pathological features, and clinical subgroups (**Figure 1G**). Notably, Kaplan-Meier analysis demonstrated significant disparities in survival prognosis among the clusters (*p* < 0.0001), with cluster C3 showing the poorest outcomes (**Figures 1H, I**). This comprehensive analysis underscores the prognostic significance of ITM and their associations with clinical features.

**Table 1:** Relationship between cluster and clinicopathological characteristics.

Characteristics	C1 (N=183)	C2 (N=77)	C3 (N=90)	Total (N=350)	P value
Sub-type					0.24
MSI-H	34 (9.71%)	12 (3.43%)	13 (3.71%)	59 (16.86%)	
MSI-L	21 (6.00%)	17 (4.86%)	13 (3.71%)	51 (14.57%)	
MSS	128 (36.57%)	48 (13.71%)	64 (18.29%)	240 (68.57%)	
Age					0.26
Mean ± SD	65.85 ± 10.81	63.86 ± 9.77	66.24 ± 10.91	65.51 ± 10.62	
Median[min-max]	68.00[35.00,90.00]	65.00[41.00,83.00]	67.00[41.00,90.00]	67.00[35.00,90.00]	
Gender					0.31
FEMALE	68 (19.43%)	30 (8.57%)	26 (7.43%)	124 (35.43%)	
MALE	115 (32.86%)	47 (13.43%)	64 (18.29%)	226 (64.57%)	
Grade					0.44
G1	3 (0.86%)	2 (0.57%)	4 (1.14%)	9 (2.57%)	
G2	63 (18.00%)	24 (6.86%)	38 (10.86%)	125 (35.71%)	
G3	111 (31.71%)	50 (14.29%)	46 (13.14%)	207 (59.14%)	
GX	6 (1.71%)	1 (0.29%)	2 (0.57%)	9 (2.57%)	
Pathologic_M					0.01
M0	164 (46.86%)	72 (20.57%)	75 (21.43%)	311 (88.86%)	
M1	7 (2.00%)	4 (1.14%)	12 (3.43%)	23 (6.57%)	
Pathologic_N					0.06
N0	53 (15.14%)	29 (8.29%)	20 (5.71%)	102 (29.14%)	
N1	46 (13.14%)	20 (5.71%)	29 (8.29%)	95 (27.14%)	
N2	39 (11.14%)	18 (5.14%)	16 (4.57%)	73 (20.86%)	
N3	41 (11.71%)	10 (2.86%)	19 (5.43%)	70 (20.00%)	
Pathologic_T					5.70E-03
T1	7 (2.00%)	2 (0.57%)	8 (2.29%)	17 (4.86%)	
T2	34 (9.71%)	22 (6.29%)	16 (4.57%)	72 (20.57%)	
T3	85 (24.29%)	35 (10.00%)	43 (12.29%)	163 (46.57%)	
T4	57 (16.29%)	18 (5.14%)	19 (5.43%)	94 (26.86%)	
Stage					0.00044
I	24 (6.86%)	10 (2.86%)	13 (3.71%)	47 (13.43%)	
II	61 (17.43%)	29 (8.29%)	18 (5.14%)	108 (30.86%)	
III	86 (24.57%)	28 (8.00%)	34 (9.71%)	148 (42.29%)	
IV	10 (2.86%)	7 (2.00%)	17 (4.86%)		

MSI-H, Microsatellite Instability-High; MSI-L, Microsatellite Instability-Low; MSS, Microsatellite Stability.

### 3.2 Potential immune-microbe interactions and molecular mechanisms

**Figure 2:**
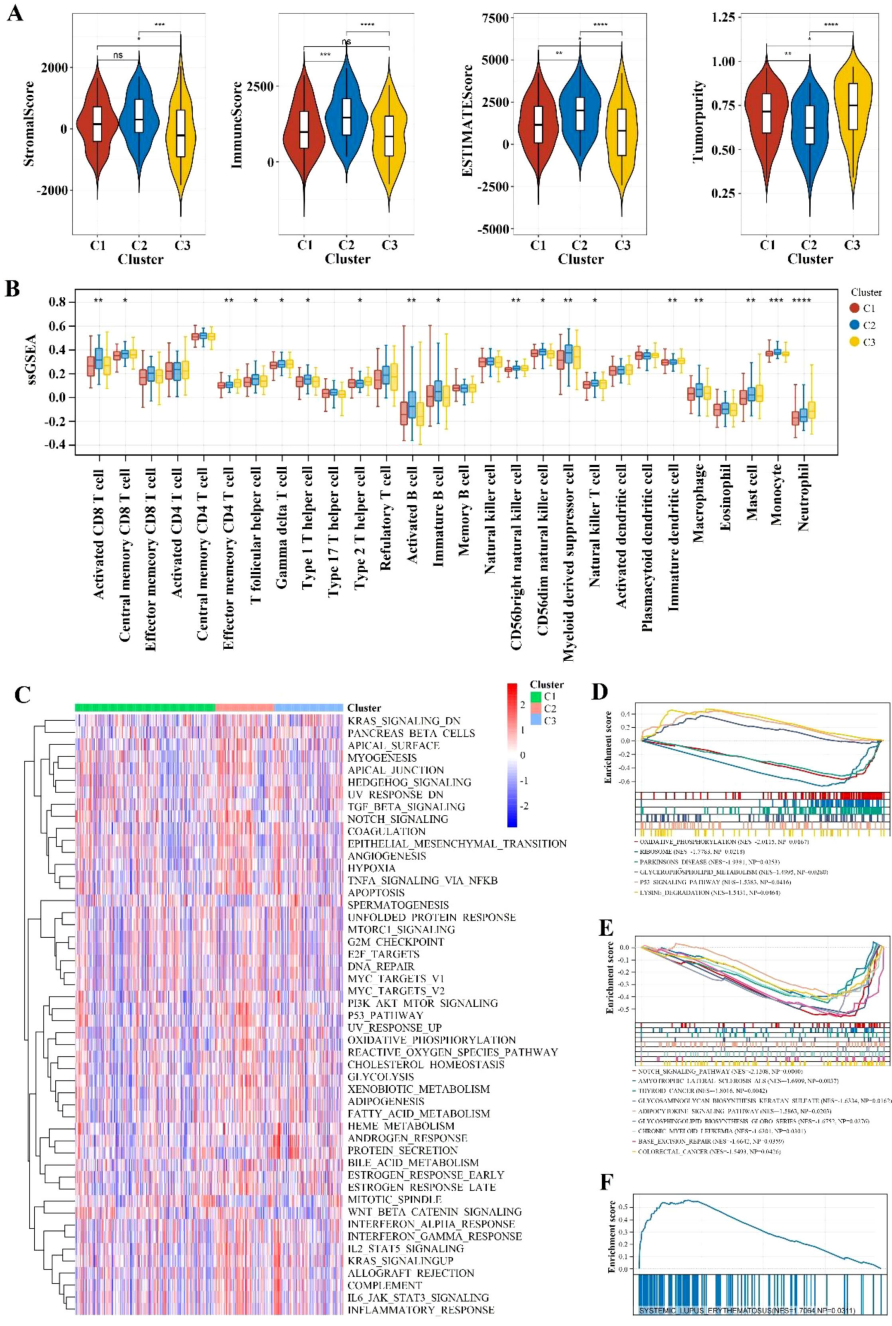
Immune infiltration and pathway enrichment analysis. **(A)** Violin plot comparing immune scores and stromal scores across different Cluster groups. ** indicates p < 0.01; *** indicates p < 0.001; **** indicates p < 0.0001; ns indicates no significance. **(B)** Comparison of immune cell types with significant differences between different Cluster groups using the ssGSEA algorithm. * indicates p < 0.05; ** indicates p < 0.01; *** indicates p < 0.001; **** indicates p < 0.0001. **(C)** Heatmap of enrichment differences for hallmark gene sets. **(D-F)** Significantly different KEGG pathways between different Cluster groups with a threshold of p < 0.05 and an absolute NES value greater than 1.

The analysis of Stromal Score, Immune Score, ESTIMATE Score, and Tumor Purity across the three clusters (C1, C2, C3) revealed that C3 is predominantly comprised of tumor cells, with significantly lower infiltration of stromal and immune cells compared to C1 (*p* < 0.05) and C2 (*p* < 0.001). Conversely, C2 exhibits greater immune cell infiltration than C1 (*p* < 0.001) (**Figure 2A**), emphasizing distinct stromal, immune, and overall microenvironmental characteristics among the clusters, which may influence gastric cancer (GC) prognosis and treatment strategies.

The ssGSEA analysis of three clusters (C1, C2, C3) revealed distinct immune cell compositions. Cluster C2 shows higher enrichment of activated CD8+ T cells (*p* < 0.01), effector memory CD4 T cells (*p* < 0.01), Type 1 T helper cells (*p* < 0.01), regulatory T cells (Tregs) (*p* < 0.01)), indicating a robust cytotoxic, helper T cell, and memory response. Cluster C1 is characterized by higher levels of activated B cells (*p* < 0.05), activated dendritic cells (*p* < 0.05), and plasmacytoid dendritic cells (*p* < 0.05), suggesting an active antigen-presenting cell response. Cluster C3 exhibits a higher presence of M2 macrophages (*p* < 0.01), indicating a potential promotion of tumor growth and metastasis (**Figure 2B**).

The variations in gene expression patterns and the enrichment of hallmark gene sets among different clusters were analyzed to promote a deeper understanding of their biological differences. As shown in **Figure 2C**, some set of genes associated with the progression of gastric cancer such as KRAS signaling and Protein secretion were up-regulated especially in cluster C3. Besides, the GSEA analysis revealed that cluster C1 exhibits significant enrichment in oxidative phosphorylation, ribosome activity, and Parkinson’s disease pathways. This reflected the cells’ elevated energy demands and rapid proliferation, which might favor the activation and expansion of immune cells. Cluster C2 is notably enriched in the systemic lupus erythematosus pathway, indicating active immune responses within the TME. Cluster C3 shows significant enrichment in the Notch signaling, which points to its underlying role in regulating the differentiation and proliferation of tumor cells and promote cancer progress (**Figures 2D–F**).

### 3.3 Identification of microbiome-associated immune genes with prognostic value

**Figure 3:**
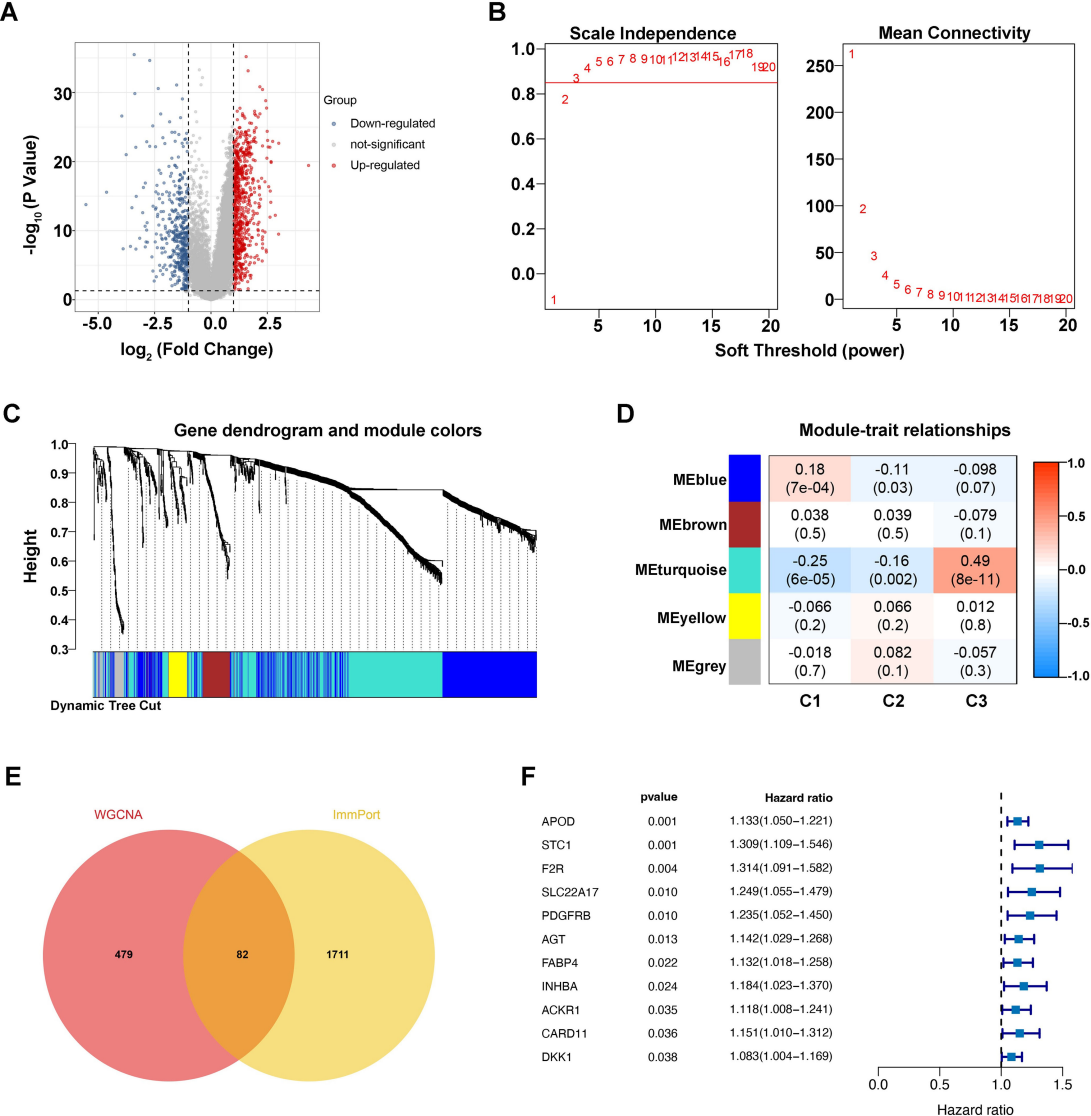
Identification of microbiome-associated immune genes with prognostic value. **(A)** Differential gene volcano plot, where blue represents downregulated genes and red represents upregulated genes. **(B)** Left: Plot for selecting the weight parameter “power” in the adjacency matrix. The x-axis represents the weight parameter “power,” and the y-axis represents the square of the correlation coefficient between log(k) and log(p(k)) in the corresponding network. A higher value of the square of the correlation coefficient indicates that the network is closer to a scale-free distribution. The red line indicates the standard line where the square of the correlation coefficient reaches 0.85. Right: Schematic diagram of the average gene connectivity under different “power” parameters in the adjacency matrix. The red line indicates the average connectivity of network nodes at the “power” parameter value selected in the left plot. **(C)** Dendrogram of module partitioning. Each color represents a different module. **(D)** Heatmap showing the correlation between each module and phenotypic traits. **(E)** Venn diagram of intersections. **(F)** Univariate Cox forest plot.

Considering the above evidence, we conducted that the different tumor-bacteria clusters were related to immune microenvironment reshaping. Therefore, we performed a comparative analysis and identified 1206 differentially expressed genes (DEGs) related to immune (**Figure 3A**). Using WGCNA with phenotypic markers C1, C2, and C3, we optimized the network’s ‘power’ parameter at 3 to meet scale-free topology conditions (**Figure 3B**). Genes with high correlations were clustered into five distinct modules. Among these, the turquoise module, consisting of 561 genes, showed the strongest correlation with phenotypic markers and was selected for further analysis as a set of intra-tumoral microbiome-related genes (**Figures 3C, D**).

To explore host-microbiome interactions, we cross-referenced immune genes from the ImmPort database with those in the turquoise module, identifying 82 immune genes closely linked to the intra-tumoral microbiome (**Figure 3E**). Survival analysis then revealed eleven significant genes, including APOD (p=0.001, HR=1.133), STC1 (p=0.001, HR=1.309), and others, as shown in **Figure 3F**, suggesting that they may be key immune gene targets associated with intratumoral microbial signature and influence gastric cancer prognosis.

### 3.4 Prognostic intratumoral microorganisms and microbial clustering analysis

**Figure 4:**
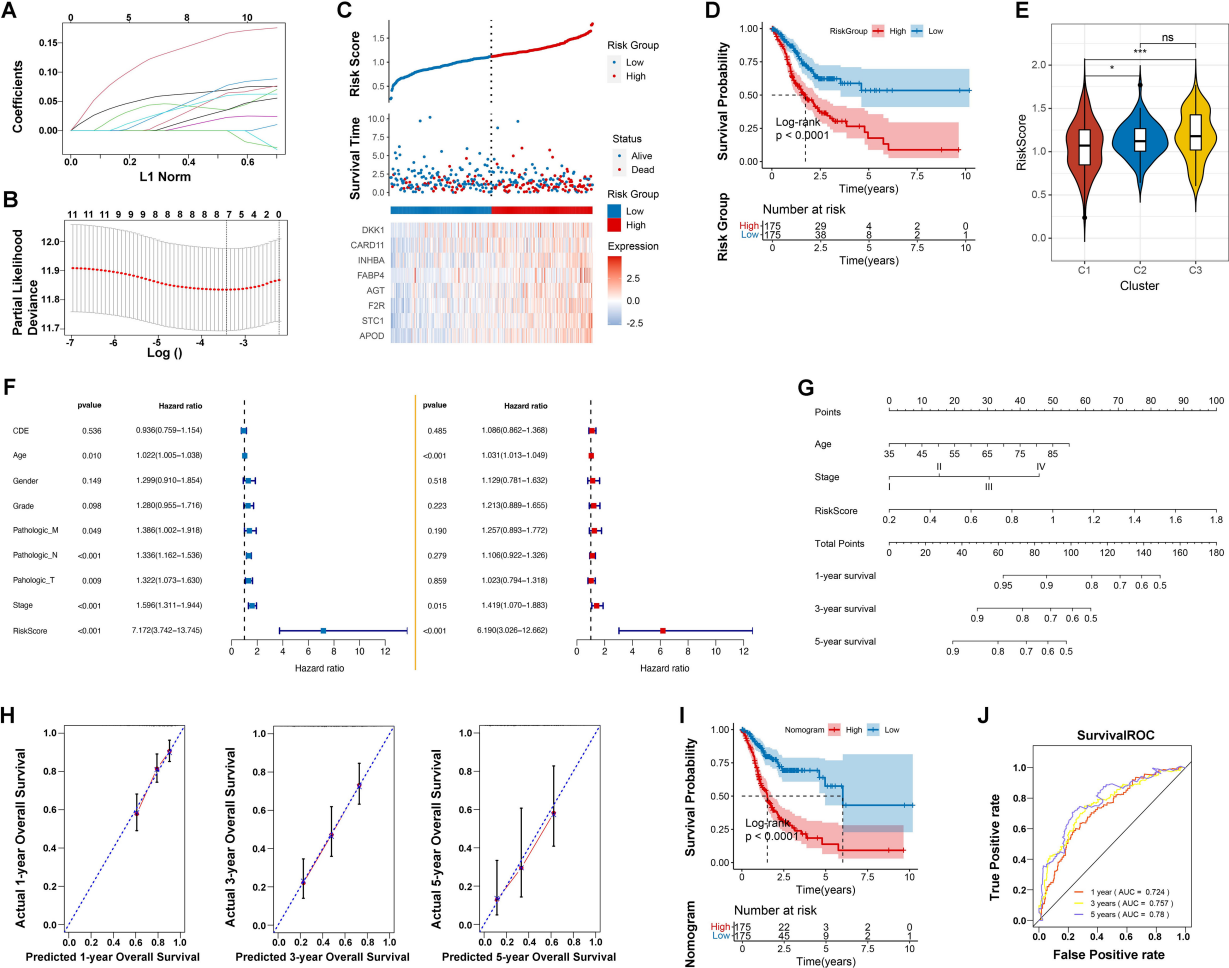
Prognostic model development and validation. **(A)** Distribution of LASSO coefficients. **(B)** Likelihood deviation of the LASSO coefficient distribution, with two vertical dashed lines representing lambda.min (left black line) and lambda.1se (right black line), respectively. **(C)** Distribution of RiskScore (top), survival time status (middle), and gene expression pattern of the model (bottom) in the TCGA training set. **(D)** KM curve for prognosis prediction based on the RiskScore model. **(E)** Differences in RiskScore distribution between different Clusters. * indicates p < 0.05; *** indicates p < 0.001; ns indicates no significance. **(F)** Forest plot of univariate and multivariate Cox regression analysis for clinical information. **(G)** Nomogram for predicting survival rates using independent prognostic factors. **(H)** Calibration plot for 1-year, 3-year, and 5-year survival predictions from the nomogram, with the x-axis representing predicted survival rates and the y-axis representing actual survival rates. **(I)** KM curve for prognosis prediction based on the nomogram model. **(J)** ROC curves for 1-year, 3-year, and 5-year predictions based on the nomogram.

Using the LASSO regression algorithm, we developed an eight-gene RiskScore model significantly associated with GC prognosis (**Figures 4A, B**): RiskScore = 0.0607 * APOD + 0.1345 * STC1 + 0.0455 * F2R + 0.0017 * AGT + 0.0082 * FABP4 + 0.0185 * INHBA + 0.0354 * CARD11 + 0.034 * DKK1.

This model effectively distinguished low-risk from high-risk patients in the TCGA training dataset, with significant survival differences (p < 0.0001). High-risk groups had a 5-year survival rate of 25%, compared to 60% in low-risk groups. The model’s predictive performance improved over time, with AUC values increasing from 0.661 at 1 year to 0.760 at 5 years (**Figures 4C, D**; **Supplementary Figure S1A**). Validation using an independent GEO dataset confirmed consistent predictive accuracy (**Supplementary Figure S1B-D**). Notably, riskscores were significantly different in distinct microbial characterization groups, especially the cluster 3 with poor microbe-associated immune infiltration obtained the elevated riskscores (**Figure 4E**). This highlights the strong correction among introtumoral microbes, immune microenvironment and our RiskScore model.

Univariate Cox regression identified Age, Pathologic M, N, and T, Stage, and the RiskScore model as significant prognostic factors (p < 0.005). Multivariate analysis confirmed Age (HR = 1.031, *p* < 0.001), Stage (HR = 1.419, *p =*0.015), and the RiskScore model (HR = 6.190, *p* < 0.001) as independent predictors, with RiskScore having the highest hazard ratios (**Figure 4F**). To enhance predictive accuracy, a Nomogram model integrating RiskScore, age, and tumor stage was constructed and validated. Kaplan-Meier survival analyses revealed significant differences between high and low-risk groups (p < 0.0001). The Nomogram’s C-index was 0.752, and ROC curves for 1-year (AUC = 0.724), 3-year (AUC = 0.757), and 5-year (AUC = 0.78) survival rates underscored the model’s robustness in long-term survival prediction, supporting its use in clinical risk assessment and personalized treatment strategies (**Figures 4H–J**).

### 3.5 Mutation status analysis and investigation of immune-microbial interactions

**Figure 5:**
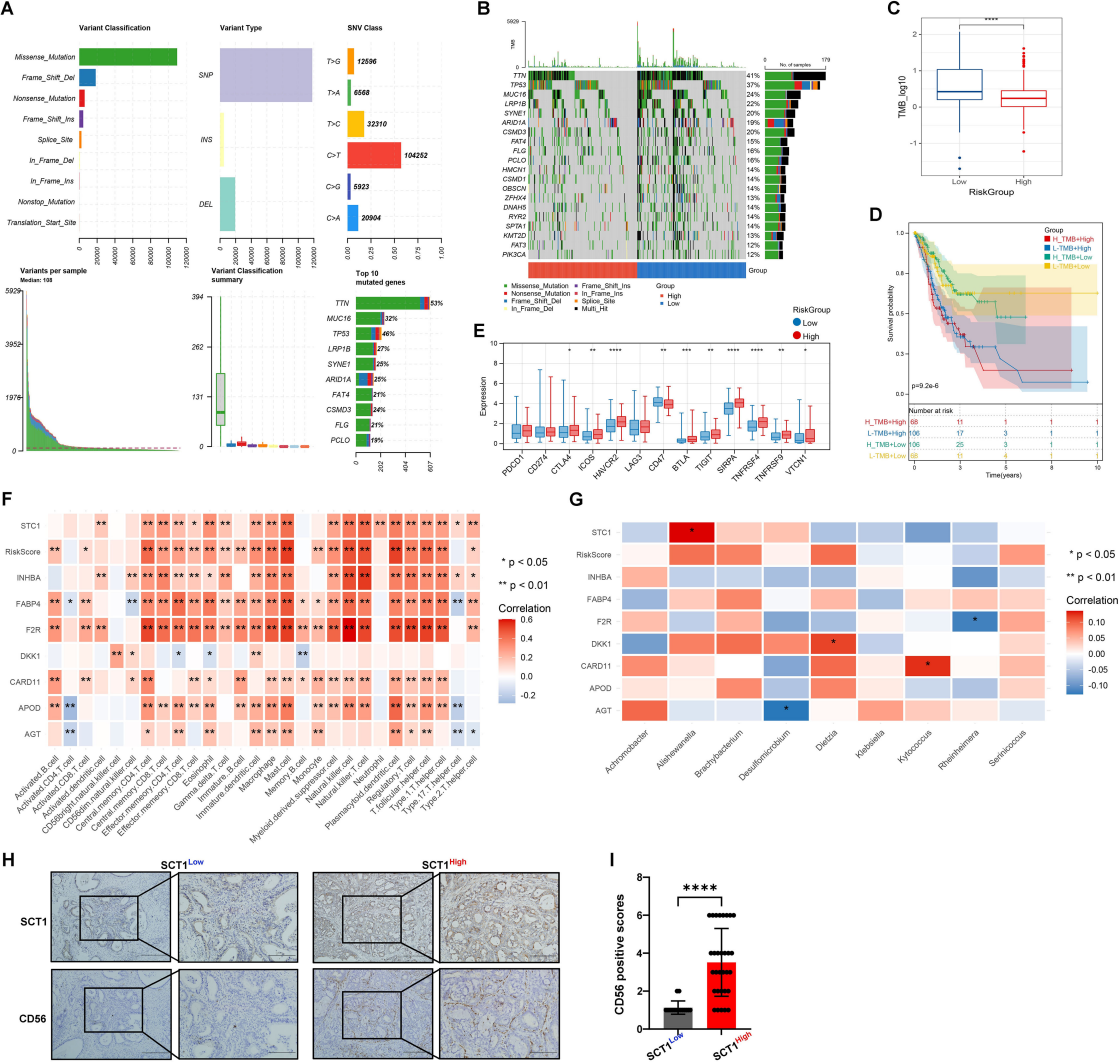
Mutation status and immune-microbial interactions analysis. **(A, B)** Mutation waterfall plots of Top20 genes in different risk groups. **(C)** Box plot of TMB differences. **(D)** Combined KM curve. **(E)** Box plot of differences in immune checkpoint expression. **(F)** and **(G)** Heatmaps of correlation between RiskScore and immune cells. **(H, I)** IHC validation shows colocalization and positive correlation of STC1 and CD56 expression in tumor tissues (* indicates p < 0.05; ** indicates p < 0.01; *** indicates p < 0.001); **** indicates p < 0.0001.

Research has demonstrated that TMB is associated with the efficacy of immunotherapy and the prognosis of various cancers (16). Therefore, this study conducted an analysis of TMB status and found a prevalence of single nucleotide polymorphisms, particularly C>T transitions, with missense mutations being the most frequent (**Figure 5A**). Notably, key genes such as TTN, MUC16, and TP53 are frequently mutated (**Figure 5B**). Additionally, a higher TMB is associated with a lower risk (**Figure 5C**). Survival analysis revealed that patients with low TMB and low risk have the highest survival probability (**Figure 5D**), indicating that TMB plays a crucial role in prognosticating patient outcomes and enhancing the accuracy of survival assessments.

**Figure 5E** reveals significant variability in RiskScores among clusters. High-risk groups exhibit higher expression levels of immune checkpoint molecules compared to low-risk groups, suggesting a potential correlation with increased disease risk. Our analysis identified significant correlation patterns between eight genes (STC1, INHBA, FABP4, F2R, DKK1, CARD11, APOD, AGT), RiskScore, immune cell types, and bacterial species. Notably, RiskScore has strong positive correlations with numerous immune cell types, such as activated CD8 T cells, regulatory T cells, and NK cells (p < 0.01), indicating its role in immune modulation. STC1 and INHBA showed strong positive correlations with Effector memory CD4 T cells (p < 0.01) and NK cells (p < 0.01), respectively. FABP4 and F2R were associated with Activated B cells and Macrophages (p < 0.05) (**Figure 5F**). Additionally, STC1 exhibited a significant positive correlation with Alishewanella (p < 0.05). DKK1 showed a significant positive correlation with Dietzia (p < 0.05), whereas AGT was significantly negatively correlated with Desulfomicrobium and F2R with Rheinheimera (p < 0.05). CARD11 positively correlated with Kytococcus (p < 0.05), and AGT showed negative correlation with Desulfomicrobium (p < 0.05) (**Figure 5G**). IHC confirmed a positive correlation between the expression of STC1 and CD56 (**Figures 5H, I**).

### 3.6 Therapeutic implications

**Figure 6:**
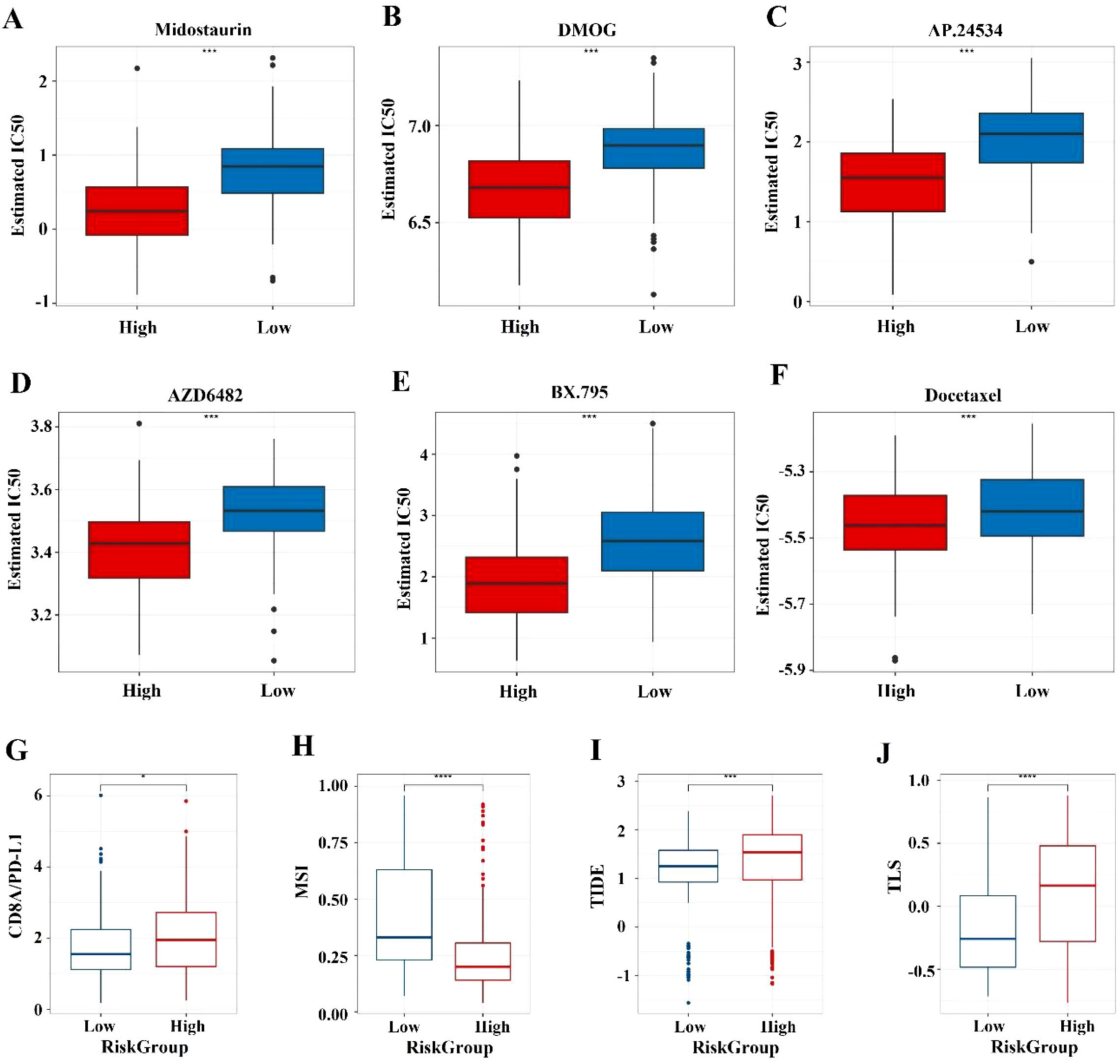
Prediction of chemotherapy sensitivity and immunotherapy response. **(A-E)** Comparison of IC50 level differences for six chemotherapy drugs across different risk groups. **(F-I)** Comparison of TIDE and immune marker scores across different risk groups. * indicates p < 0.05; *** indicates p < 0.001; **** indicates p < 0.0001.

For the high-risk group, the top five drugs with highest sensitivity are: Midostaurin (*p* = 2.72E-21), AP.24534 (*p* = 2.88E-20), DMOG (*p* = 7.68E-20), AZD6482 (*p* = 1.41E-18), and BX.795 (*p* = 1.82E-17) (**Figures 6A–E**). Common gastrointestinal cancer drugs show no significant difference, except Docetaxel (*p* = 1.05E-05) (**Figure 6F**). The high-risk group has a significantly higher median TIDE score (*p* < 0.001) (**Figure 6I**), indicating greater immune dysfunction and exclusion. The high-risk group also shows higher CD8A/PD-L1 ratios (*p* < 0.05) and TLS scores (*p* < 0.0001) (**Figures 6G, J**), but lower MSI scores (*p* < 0.0001) (**Figure 6H**) compared to the low-risk group. No significant differences in CYT values.

### 3.7 Single-cell analysis

**Figure 7:**
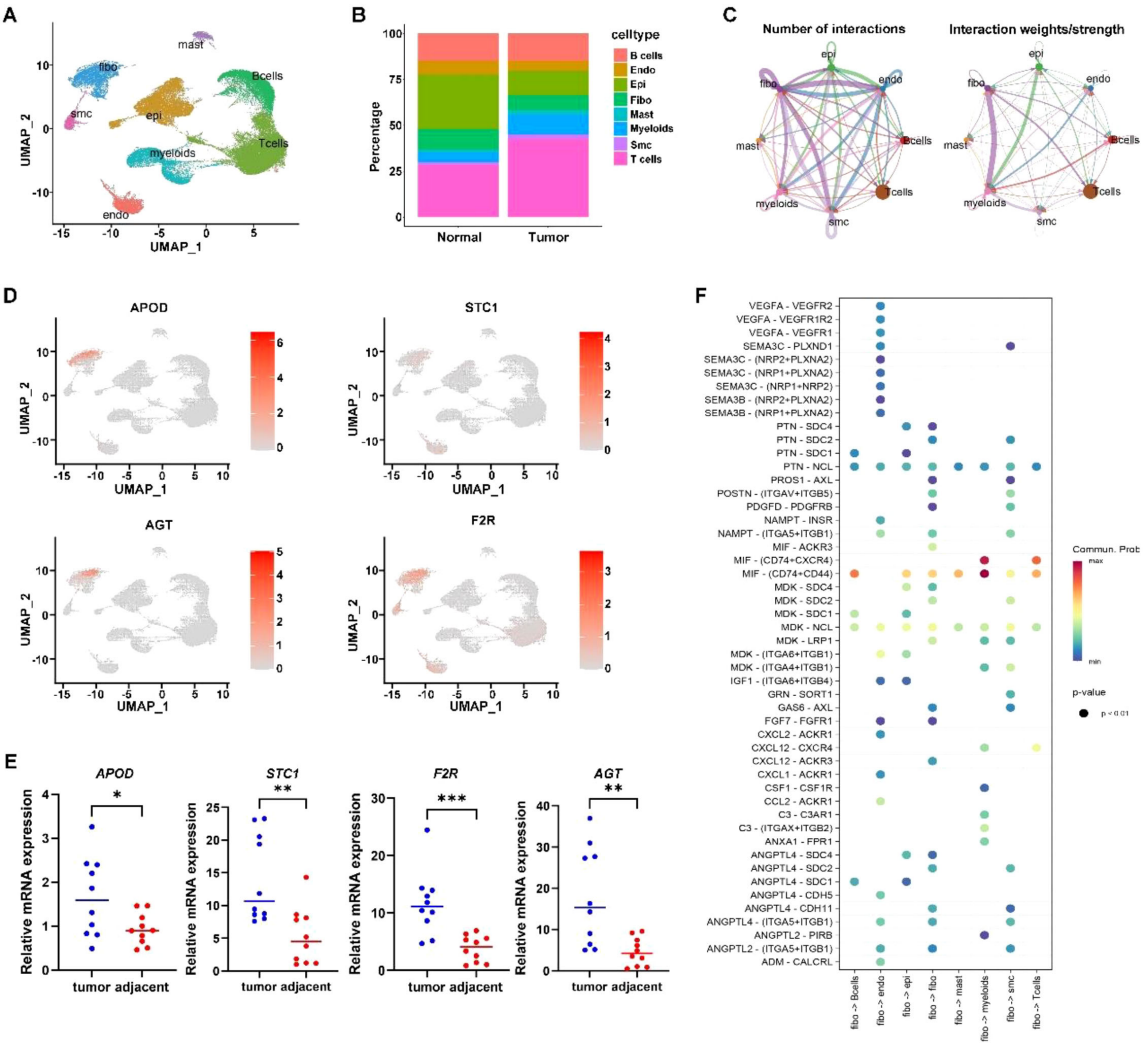
Single cell analysis. **(A)** Cell annotation plot. **(B)** Cell distribution ratio plot. **(C)** shows the number of ligand-receptor pairs and communication probability among all cell populations across samples; different colors in the outer circle represent different cell populations, and the size indicates the number of ligand-receptor pairs in the population. A larger circle indicates a higher ratio of ligand-receptor pairs between cells. **(D)** Expression of signature genes in single cells. **(E)** qPCR confirmation of signature gene expression in tumor tissues. **(F)** In the bubble plot, the x-axis represents cell pairs, with colors distinguishing samples; the y-axis represents ligands and receptors. The size of the bubble indicates the p-value, with smaller p-values corresponding to larger bubbles. The color represents the magnitude of communication probability. * indicates p < 0.05; ** indicates p < 0.01; *** indicates p < 0.001.

Finally, based on the annotation of 31 cell clusters, we found significant differences in the proportions of 8 cell types between tumor and normal tissues (**Figures 7A, B**). Further analysis revealed that APOD, STC1, F2R, and AGT genes are mainly expressed in tumor-associated fibroblasts (**Figure 7D**). qPCR confirmed their higher expression in cancer tissues (**Figure 7E**). Cell communication analysis showed strong interactions between fibroblasts and other cells (**Figure 7C**), with ligand-receptor pairs like MIF - (CD74+CD44) activated (**Figure 7F**). Thus, we speculate that the influence of the ITM on immune gene expression differences associated with prognosis is probably mediated by tumor-associated fibroblasts.

To further validate the presence and activation of cancer-associated fibroblasts, we examined the expression of the CAF markers α-SMA and vimentin. qRT-PCR analysis revealed that both ACTA2 and VIM were significantly upregulated in gastric cancer tissues compared to adjacent normal tissues (*p* < 0.01, **Supplementary Figure S2**). These findings confirm the activation status of fibroblasts in GC tissues and support the role of tumor-associated fibroblasts in the immune-microbiome interaction framework described in this study.

## 4 Discussion

This study enhances our understanding of the prognostic significance of intratumoral microorganisms and their interactions with immune responses in GC. Our comprehensive analysis identified 229 differentially expressed genera, with nine bacterial genera significantly associated OS, emphasizing the prognostic value of the tumor-associated microbiome. These findings align with recent studies highlighting the crucial role of the microbiome in cancer prognosis and therapy response (17–19). Our research indicates that Serinicoccus, Desulfomicrobium, Brachybacterium, Dietzia, Alishewanella, Kytococcus, and Rheinheimera are associated with increased risk, while Klebsiella and Achromobacter are correlated with decreased risk. This is different from a recently reported literature, which identified five bacterial genera associated with poor prognosis of GC but did not discover protective bacterial genera that reduce risk (20). Moreover, upon closer comparison, the harmful bacterial genera are also different. This suggests that there may be significant variations in the ITM of GC across different regions and populations, highlighting the need for further research.

Further analysis revealed three distinct microbial clusters with unique survival outcomes and immune profiles, reinforcing the heterogeneity of GC. This echoes with recent findings that microbiome composition can stratify patients into subgroups with different prognoses (20, 21). Specifically, Cluster C1 with the best prognosis is distinguished by elevated levels of activated B cells, activated dendritic cells, and plasmacytoid dendritic cells, suggesting an active antigen-presenting cell response. This phenotype is reminiscent of the robust immune activation observed in autoimmune diseases or during acute immune challenges (22). The enrichment of oxidative phosphorylation and ribosome activity in C1 supports heightened metabolic activity and protein synthesis (23), suggesting involvement in rapid immune responses. C2 demonstrates robust cytotoxic, helper T cell, and memory responses, aligning with the importance of these cells in eliminating infected or transformed cells (24, 25). C3, with poor prognosis, is characterized by higher M2 macrophages and neutrophils, indicating potential tumor promotion (26–28). Compared to C2, C3 shares six high-abundance bacterial genera but lacks Kytococcus, Klebsiella, and Achromobacter. This suggests these three may be associated with better immune infiltration and prognosis, requiring further evidence.

Immune-microbe interaction analysis emphasized the complex relationships within the TME. The positive correlation between certain microbial genera and immune cell infiltration supports the hypothesis that the microbiome can modulate immune responses (29, 30). For instance, high levels of Bifidobacterium were associated with increased T-cell infiltration and improved survival, suggesting its potential role in immunotherapy enhancement (31). Cluster 3 with poor microbe-associated immune infiltration had higher risk scores, indicating a strong correlation between intratumoral microbes, immune microenvironment, and our RiskScore model. This model, based on microbiome-related immune genes, functioned as an independent prognostic factor and, combined with clinical factors, formed a Nomogram model. Its predictive performance improved over time, supporting its use in clinical risk assessment and personalized treatment strategies. This aligns with the interest in developing microbiome-based biomarkers for cancer prognosis (32, 33).

High TMB has been associated with better responses to immunotherapy due to the higher neoantigen load. Studies have indicated that TMB can predict survival after immunotherapy across multiple cancer types (16). Our mutation analysis demonstrated the genetic variability impacting TMB and survival. The L_TMB+High risk group have the lowest survival probability over time. This analysis demonstrates that integrating TMB into prognostic models provides a more accurate assessment of patient survival. Immune checkpoint molecule expression analysis indicated immune evasion mechanisms in high-risk groups, which is crucial for identifying patients who may benefit from immune checkpoint inhibitors (34).

Therapeutic implications highlight the importance of personalized treatment based on microbiome and immune profiles. The microbiome’s potential to modulate responses to chemotherapy, radiation, and immunotherapy enhances treatment efficacy (35, 36). Our study shows no significant differences in commonly used chemotherapy drugs for gastrointestinal cancers between groups, except for Docetaxel. However, notable differences emerged in immunological markers between High-risk and Low-risk groups, suggesting potential differences in immunotherapy responsiveness. This indicates that GC can be treated by regulating microorganism-gene-immunity interactions. Modulating the microbiome through probiotics, prebiotics, or fecal microbiome transplantation offers promising new therapeutic strategies (37, 38).

Although Klebsiella and Achromobacter are occasionally implicated in opportunistic infections, especially in immunocompromised hosts or hospital environments, their role within the gastric tumor microenvironment appears to be context-dependent and potentially beneficial (26). In our study, elevated levels of these genera correlated with improved prognosis and enhanced immune cell infiltration, particularly CD8+ cytotoxic T cells and helper T cells in Cluster C2. This suggests a potential immunostimulatory role that may help restrain tumor progression. While the precise mechanisms remain under investigation, our analysis identified significant correlations between these microbes and host immune genes such as CARD11 and AGT, which are involved in T-cell signaling and renin-angiotensin regulation, respectively. These interactions may facilitate a more immunologically active tumor microenvironment that limits cancer progression. Therefore, Klebsiella and Achromobacter may exert protective effects not through direct bactericidal action, but by modulating host immunity. Further studies, including gnotobiotic models and functional validation, will be essential to elucidate their exact roles and therapeutic potential in GC.

In addition, although H. pylori infection plays a central role in the initiation of gastric cancer, its presence is often diminished or absent in advanced-stage tumor tissues. This is likely due to progressive mucosal atrophy and tumor-induced environmental changes that no longer support H. pylori colonization. Our cohort consisted predominantly of late-stage GC samples, consistent with previous findings that show a marked decline in H. pylori detection at later disease stages. These observations suggest a dynamic shift in the gastric microbial community during tumor progression, in which other microbial species may become more dominant and influence prognosis and immune modulation.

The integration of bioinformatics validation through qPCR and IHC adds robustness to our findings, yet further large-scale validation is warranted. Our findings lay the groundwork for integrating microbiome and immune signatures into clinical practice, potentially enhancing prognostic accuracy and patient outcomes in GC. Future research should prioritize longitudinal studies to understand microbiome-TME interactions (7, 39)and mechanistic studies to elucidate the impacts of specific microbes on cancer progression (40, 41). In particular, the mechanism of tumor-associated fibroblasts in regulating intratumoral bacteria-related immunity merits further investigation. However, limitations exist, including potential biases in publicly available data and bioinformatics tools, as well as the need for larger cohorts and longitudinal data for further validation and causal inference.

The identification of nine intratumoral microbial genera associated with patient prognosis provides novel insight into the microbial component of the gastric tumor microenvironment. Notably, genera such as Klebsiella and Achromobacter were enriched in tumor samples with better survival and stronger immune infiltration, suggesting a potential immunomodulatory or anti-tumor role. Conversely, risk-associated genera including Desulfomicrobium and Dietzia were more prevalent in patients with poorer prognosis, raising the possibility that they may contribute to immunosuppression or tumor-promoting inflammation. Although direct causality cannot be established from our data alone, these microbial signatures are consistent with emerging evidence that non-Helicobacter pylori bacteria can shape local immune responses and influence tumor behavior. Thus, the prognostic associations observed in our study support the concept that the intratumoral microbiome is not merely a passive bystander but may represent an integral component of the gastric cancer ecosystem. These findings warrant further mechanistic investigations and highlight the potential for microbial profiling to complement existing clinical parameters in prognostic assessment.

The observed immune heterogeneity among microbiota-based clusters supports the notion that distinct microbial communities may shape the tumor immune microenvironment in gastric cancer. Specifically, the enrichment of Klebsiella and Achromobacter in Cluster C2 coincided with increased infiltration of activated CD8&^+^ T cells and helper T cells, suggesting a possible role for these genera in promoting anti-tumor immune responses. In contrast, Clusters enriched with genera such as Desulfomicrobium and Dietzia exhibited immunosuppressive features, including elevated M2 macrophages and reduced T cell activity. These patterns underscore the potential of intratumoral microbes to modulate immune surveillance and tumor progression.

In summary, our study highlights the significant role of microbiome-related immune gene signatures in GC prognosis, offering a novel approach for risk stratification and personalized therapy. These findings, supported by recent literature, validate the potential clinical application of microbiome and immune profiling in improving patient outcomes.

## Data Availability

The original contributions presented in the study are included in the article/**Supplementary Material**, further inquiries can be directed to the corresponding author/s.
