# Correction: Identification of prognosis and therapy related intratumoral microbiome and immune signatures in gastric cancer

**DOI:** 10.3389/fimmu.2025.1670563

**Published:** 2025-08-12

**Authors:** Peng Jin, Xiaoyan Ji, Jingchao Bai, Wei Su, Bin Ke, Yong Liu, Bin Wang

**Affiliations:** ^1^ Department of Gastric Surgery, Tianjin Medical University Cancer Institute and Hospital, National Clinical Research Center for Cancer, Key Laboratory of Cancer Prevention and Therapy, Tianjin Key Laboratory of Digestive Cancer, Tianjin’s Clinical Research Center for Cancer, Tianjin, China; ^2^ Department of Emergency Ward, First Teaching Hospital of Tianjin University of Traditional Chinese Medicine, National Clinical Research Center for Chinese Medicine Acupuncture and Moxibustion, Tianjin, China; ^3^ Department of Gastrointestinal Cancer Biology, Tianjin Medical University Cancer Institute and Hospital, Key Laboratory of Cancer Immunology and Biotherapy, National Clinical Research Center for Cancer, Tianjin Medical University Cancer Institute and Hospital, Tianjin, China; ^4^ Department of Endoscopy, Tianjin Medical University Cancer Institute and Hospital, National Clinical Research Center for Cancer, Key Laboratory of Cancer Prevention and Therapy, Tianjin’s Clinical Research Center for Cancer, Tianjin, China; ^5^ Department of Pancreatic Cancer, Tianjin Medical University Cancer Institute and Hospital, National Clinical Research Center for Cancer, Tianjin Key Laboratory of Digestive Cancer, Key Laboratory of Cancer Prevention and Therapy, Tianjin, China

**Keywords:** gastric cancer, intratumoral microbiome, prognostic biomarkers, therapeutic responses, drug sensitivity, comprehensive analysis

In the published article, there was an error in the **Funding** statement. The funder “Tianjin Key Medical Discipline (Specialty) Construction Project, TJYXZDXK-009A” was erroneously omitted. The correct **Funding** statement appears below.

“The author(s) declare that financial support was received for the research and/or publication of this article. This work was supported by grants from the Tianjin Key Medical Discipline (Specialty) Construction Project (TJYXZDXK-009A), Introduction of Talents and Doctoral Startup Fund (B2301 and B2320), The China Postdoctoral Science Foundation (2023M742618) and China National Postdoctoral Program for Innovative Talent (BX20240254).”

The original article has been updated.

