# Supplementary material for: Identification of prognosis and therapy related intratumoral microbiome and immune signatures in gastric cancer
Source: Front Immunol. 2025 Jul 3;16:1622959. doi: 10.3389/fimmu.2025.1622959 (PMC12267262; doi:10.3389/fimmu.2025.1622959)
Supplement: Supplementary file 1 [file DataSheet1.pdf]

Supplementary Material

Supplementary Figures

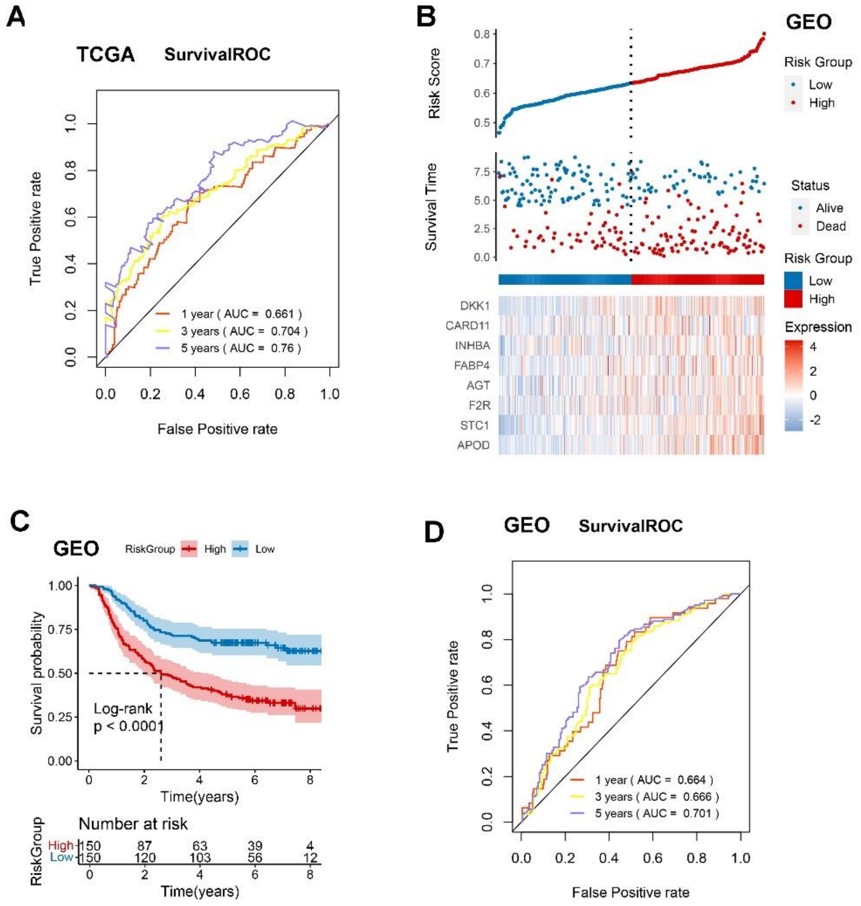

Supplementary Figure S1. (A) ROC curves for 1-year, 3-year, and 5-year predictions based on TCGA data set. B-D Analysis in the GEO validating set. (B) Distribution of RiskScore (top), survival time status (middle), and gene expression pattern of the model (bottom). (C) KM curve for prognosis

prediction based on the RiskScore model. (D) ROC curves for 1-year, 3-year, and 5-year predictions based on GEO data set.

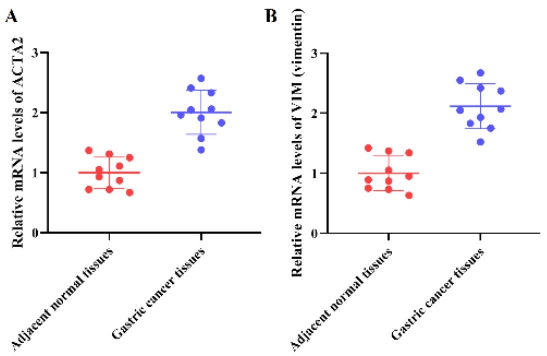

Supplementary Figure S2. ACTA2 and VIM was upregulated in GC tissues. qRT-PCR analysis of ACTA2 (encoding α-SMA) and VIM (encoding vimentin) in 10 paired gastric cancer (GC) and adjacent normal tissues. Both genes were significantly upregulated in tumor tissues.
